# Combination of Vitamin C and Curcumin Safeguards Against Methotrexate-Induced Acute Liver Injury in Mice by Synergistic Antioxidant Effects

**DOI:** 10.3389/fmed.2022.866343

**Published:** 2022-04-14

**Authors:** Dhekra Hasan Khudhair, Ali I. Al-Gareeb, Hayder M. Al-kuraishy, Aya H. El-Kadem, Engy Elekhnawy, Walaa A. Negm, Sameh Saber, Simona Cavalu, Adrian Tirla, Saqer S. Alotaibi, Gaber El-Saber Batiha

**Affiliations:** ^1^Department of Clinical Pharmacology and Medicine, College of Medicine, University of Al-Mustansiriyah, Baghdad, Iraq; ^2^Department of Pharmacology, Faculty of Pharmacy, Tanta University, Tanta, Egypt; ^3^Pharmaceutical Microbiology Department, Faculty of Pharmacy, Tanta University, Tanta, Egypt; ^4^Department of Pharmacognosy, Faculty of Pharmacy, Tanta University, Tanta, Egypt; ^5^Department of Pharmacology, Faculty of Pharmacy, Delta University for Science and Technology, Gamasa, Egypt; ^6^Faculty of Medicine and Pharmacy, University of Oradea, Oradea, Romania; ^7^Department of Biotechnology, College of Science, Taif University, Taif, Saudi Arabia; ^8^Department of Pharmacology and Therapeutics, Faculty of Veterinary Medicine, Damanhour University, Damanhour, Egypt

**Keywords:** liver, oxidative stress, antioxidants, vitamin C, curcumin, Methotrexate

## Abstract

Methotrexate (MTX), an antineoplastic and immunosuppressive drug, widely used in the treatment of different types of cancers and the management of chronic inflammatory diseases. However, its use is associated with hepatotoxicity. Vitamin C (VC) and curcumin (CUR) exhibit anti-inflammatory and antioxidant effects. Thus, we aimed to investigate the potential hepatoprotective effects of VC and CUR pretreatment alone and in combination against MTX-induced hepatotoxicity. Albino mice were randomly divided into 7 groups: the control group, which received only normal saline; MTX group; VC group, pretreated with VC (100 or 200 mg/kg/day orally) for 10 days; CUR group, pretreated with CUR (10 or 20 mg/kg/day orally); and combination group, which received VC (100 mg/kg) and CUR (10 mg/kg). MTX was administered (20 mg/kg, intraperitoneally) to all the groups on the tenth day to induce hepatotoxicity. Forty eight hours after MTX administration, the mice were anesthetized. Blood samples were collected, the liver was removed for biochemical analysis, and a part of the tissue was preserved in formalin for histopathological analysis. The results indicated that pretreatment with a combination of VC and CUR induced a more significant decrease in the serum levels of alanine transaminase, aspartate transaminase, alkaline phosphatase, and lactic dehydrogenase and a significant increase in the tissue level of superoxide dismutase and glutathione; furthermore, it induced a significant decrease in malondialdehyde levels and improvement in histopathological changes in the liver tissues, confirming the potential hepatoprotective effects of the combination therapy on MTX-induced liver injury. To conclude, MTX-induced hepatotoxicity is mediated by induction of oxidative stress as evident by increased lipid peroxidation and reduction of antioxidant enzyme activity. Pretreatment with VC, CUR or their combination reduces the MTX-induced hepatotoxicity by antioxidant and anti-inflammatory effects. However, the combined effect of VC and CUR provided a synergistic hepatoprotective effect that surpasses pretreatment with CUR alone but seems to be similar to that of VC 200 mg/kg/day. Therefore, VC and CUR combination or a large dose of VC could be effective against MTX-induced hepatotoxicity. In this regard, further studies are warranted to confirm the combined hepatoprotective effect of VC and CUR against MTX-induced hepatotoxicity.

## Introduction

The liver is the principal organ for the metabolism and detoxification of different drugs and xenobiotics (1). Liver injury induced by drugs is a major clinical problem, especially acute liver injury, which is the main cause of drug withdrawal or restricted use after marketing (2, 3). As a result, investigating safe natural products to provide liver protection has become essential (1).

Methotrexate (MTX), an antineoplastic and immunosuppressive drug, is a folate analog widely used in the treatment of different types of cancers and the management of chronic inflammatory diseases (4, 5). It has been reported that 20–30% of patients stop MTX treatment during the first year of therapy because of intolerable side effects. MTX supposedly induces acute liver injury via oxidative stress, nitrosative stress, inflammation, apoptosis, and necrosis (6). Oxidative stress and inflammation work together to induce the apoptosis of hepatocytes (7, 8).

Several studies have reported that the use of liver protective drugs, such as sitagliptin, protect the liver from MTX-induced liver injury (9). Melatonin has also been shown to have a hepatoprotective effect against MTX-induced hepatotoxicity via its antioxidant and free radical-scavenging mechanism (10). Moreover, natural and herbal products, such as CUR, have been reported to have liver protective effects against MTX toxicity via their antioxidant property (11, 12). CUR hepatoprotective effects against MTX-induced hepatotoxicity have been evaluated in Iraqi white domestic rabbits (13).

CUR exhibits anti-inflammatory, antioxidant, antimicrobial, and anticancer effects; produces hepatic and nephroprotective effects; suppresses thrombosis; protects from myocardial infarction; and has hypoglycemic and anti-rheumatic effects. It is pharmacologically safe and has been listed as safe by the United States Food and Drug Administration (FDA). Additionally, it has been reported to have good tolerability and safety profiles in clinical trials even at doses of up to 12,000 mg/day (14, 15). The antioxidant mechanisms of CUR include scavenging of free radicals, including reactive oxygen species (ROS) and reactive nitrogen species (16).

Vitamin C (VC) is considered a circulating antioxidant and an anti-inflammatory and immune-modulating agent; it acts as a cofactor for essential mono-oxygenase and dioxygenase enzymes (17). As a result, VC exhibits a pleiotropic effect (18).

The anti-inflammatory effect of VC involves decreasing the level of C-reactive protein, which is an acute-phase protein (19). The inhibition of nuclear factor (NF)-κB decreases proinflammatory mediator levels (17). Different studies have demonstrated that VC has hepatoprotective properties. This effect is attributed to its potent antioxidant property and has been investigated *in vitro* and *in vivo* (mainly animal studies). A study on carbon tetrachloride-induced liver injury in rats reported that vitamin C normalizes the levels of alanine transaminase (ALT), aspartate transaminase (AST), alkaline phosphatase (ALP), blood hydroperoxide, and malondialdehyde (MDA) (20).

Based on previous reports, this study mainly aimed to evaluate the potential beneficial effects of the combination of CUR and VC on attenuating MTX-induced acute hepatotoxicity in mice.

## Materials and Methods

The present study was conducted at the Department of Pharmacology, College of Medicine, Al-Mustansiriyah University, and the Iraqi Center for Cancer and Medical Genetic Research, Baghdad-Iraq, from November 12, 2020, to June 1, 2021. The work commenced following approval of the ethics committee of the Department of Pharmacology, College of Medicine, Al-Mustansiriyah University. Albino Swiss mice were used for this experimental study.

### Drugs, Chemicals, and Kits

CUR was purchased from America Medic and Science (United States), vitamin C from UNIPHAR (EG), and MTX from Kocak Pharma (Turkey). All other solvents, chemicals, and kits used were purchased from Merck (United States).

### Study Design

Forty-nine Swiss albino female mice were used in this study; some of these animals were obtained from the same center, and others were obtained from the National Center for Drug Control and Research. The mice were aged 3–4 months, and their body weight was 30–40 g. They were isolated, with 7 mice per sterilized large cage, and placed at a temperature of 22–25°C with artificial 12/12 light–dark cycle and located away from noise. They were left for 1 week without any intervention for adaptation and with free access to normal mouse pellets and water. Human care for the animals was administered according to the guide and care of laboratory animals. Hepatotoxicity was induced in mice according to a previously described procedure; the dose and route of the administration of CUR were determined based on a previous study (21). The doses and route of administration of VC were determined based on a previous study (22).

After the acclimatization period of 1 week, the mice were randomly divided into 7 groups, each group with 7 mice. The duration of the study was 10 day, the intervention drugs were given from 1 to 10, on the 10th day a single MTX 20 mg/kg was give IP. Following 48 h, the mice were anesthetized and blood sample was collected from the heart of each mouse. Group 1 (control group): the mice received 0.5 mL/day of distilled water by oral gavage for 10 days. Group 2 (MTX group): the mice received a single intraperitoneal (i.p.) injection of MTX (20 mg/kg) on the tenth day of the experiment. Group 3: the mice were orally supplemented with CUR at a dose of 10 mg/kg/day by oral gavage for 10 days and MTX at the same dose as that given to group 2 on the tenth day. Group 4: the mice were orally supplemented with CUR at a dose of 20 mg/kg/day by oral gavage and on the tenth day of the experiment, they received MTX. Group 5: the mice were orally supplemented with VC at a dose of 100 mg/kg/day by oral gavage and on the tenth day of the experiment, they received MTX.Group 6: the mice were orally supplemented with VC 200 mg/kg/day by oral gavage and on the tenth day, they received MTX. Group 7: The mice were orally supplemented with CUR 10 mg/kg/day by oral gavage and VC at a dose of 100 mg/kg/day, and on the tenth day, they received MTX.

### Sample Collection

Forty-eight hours after the MTX injection, chloroform was used for inducing anesthesia. Then, blood was collected from the heart of the mouse. The blood was centrifuged for 5 min at 3,000/rpm at room temperature. The supernatant layer (serum) was isolated and frozen at –20°C to be assessed later.

### Tissue Collection

After collecting the blood sample, the animals were euthanized, and the liver was separated and washed with distilled water. A tissue slice was obtained, isolated in a plain tube, and washed in a 0.01 M monophosphate buffer solution. Then, a tissue protein extraction reagent was added in a proportion of 1 g for 5–10 mL and mixed in ice water. After blending, the mixture was centrifuged for 10 min at 5,000/rpm, and the resulting supernatant was frozen at –20°C to be assessed later.

### Determination of Hepatic Malondialdehyde

MDA and superoxide dismutase (SOD) levels in the liver tissue were estimated using an enzyme-linked immunosorbent assay (ELISA) kit (MyBioSource, United States).

### Determination of Hepatic Reduced Glutathione (GSH)

Reduced glutathione levels in hepatic tissue were assessed using the ELISA technique (MyBioSource, United States). The principle of the technique involved the utilization of the double-sandwich ELISA technique; the ELISA plate contains an antibody specific to mouse GSH monoclonal and polyclonal antibodies and biotin, which detects the antibodies.

### Determination of Lactate Dehydrogenase

A mouse Lactate Dehydrogenase (LDH) ELISA kit was designed for the determination of mouse LDH levels and is used only for research (MyBioSource, United States) involved the competitive enzyme immunoassay technique utilizing an anti-LDH antibody and an LDH–horseradish peroxidase conjugate. The procedures were performed according to the manufacturer’s protocol.

### Determination of Alanine Transaminase, Aspartate Transaminase, and Alkaline Phosphatase Levels

These biomarkers were measured using the automated device Flexor-EL80 (Vitalab, South Africa).

### Assessment of Histopathological Changes

The liver tissue samples, which were obtained from the experimental mice, were immersed in NBF for 24–48 h for fixation. Then, the liver tissues were processed using an automated tissue processor. In this study, a traditional processing procedure (the paraffin-embedded method) was used to prepare liver tissues for a microscopic examination. After tissue processing, newly prepared liquid paraffin was used for preparing a liver tissue block, and the paraffin-embedded tissue block was kept on a cold plate at 4°C overnight using the same instrument. The paraffin-embedded tissue block was cut into 5-μm sections using a microtome (LEICA RM2245, Germany) and then stained with hematoxylin and eosin. Then, a light microscopic examination was performed blindly.

In this experimental study, experimental replicates were done twice for assessment of biochemical variables including ALT, AST, ALP, LDH, GSH, SOD, and MDA levels in both serum and tissues to excluded bias and experimental errors.

### Scoring System for Histopathological Changes

In this study, liver histopathological changes were microscopically assessed by a qualified pathologist and evaluated by ranking tissue lesion severity from 0 to 3 depending on the degree of changes as follows (23)*:*

• (–) No pathological changes;
• (+ \–) Very mild histopathological changes in less than 5% of the field;
• (+) Mild histopathological changes in less than 20% of the field;
• (++) Moderate histopathological changes in 20–60% of the field;
• (+++) Severe histopathological changes in more than 60% of the field.

**FIGURE 1:**
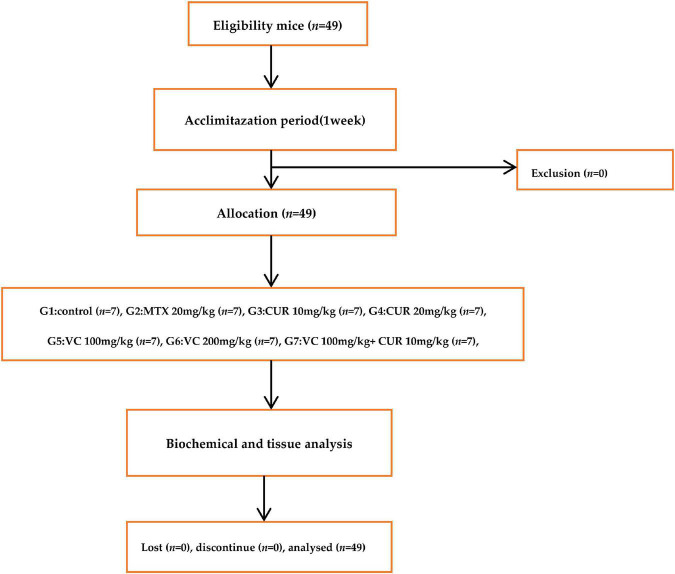
Consort-flow diagram of the study.

The study design of this experimental study as well as steps that were performed is summarized in a consort-flow diagram (Figure 1).

### Statistical Analysis

The Statistical Package for the Social Science software (version 16) was used for data analysis. The data obtained in this study were presented as mean ± standard deviation. One-way analysis of variance with *post hoc* multiple comparisons was used to investigate the significance of differences among the different groups. The probability (p) was considered significant when the value was less than 0.05.

## Results

### Effects of Treatment on Oxidative Stress Markers

**FIGURE 2:**
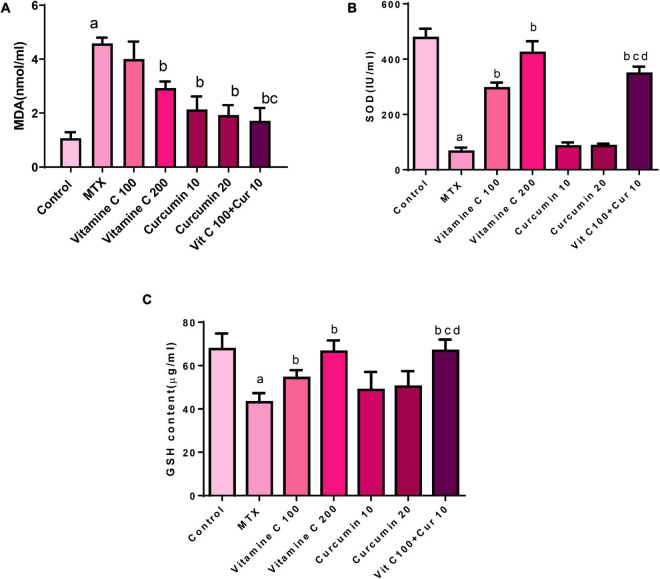
Effects of vitamin C and curcumin pretreatment alone and in combination on **(A)** MDA tissue level, **(B)** SOD activity, and **(C)** GSH tissue level (a) MTX compared to the control *P* < 0.05, (b) vitamin C or curcumin compared to MTX *P* < 0.05, (b,c) combination VC and CUR compared to VC or CUR alone *P* < 0.05, (b–d) combination VC and CUR compared to VC or CUR alone *P* < 0.

Treating the mice with single-dose MTX (20 mg/kg i.p.) resulted in a prominent oxidative stress status in the hepatic tissue, which was manifested by a significant increase in the MDA level (328.03%) compared with that in the control group (Figure 2A). Similarly, the SOD (86.269%) and GSH (36.256%) levels also significantly decreased compared with those in the control group (Figure 2B). The MDA level in the group pretreated with 100 mg/kg/day of VC was mildly decreased (12.66%) compared with that in the MTX group, and this decrease was statistically insignificant. The MDA level in the group pretreated with 200 mg/kg/day of VC was significantly decreased (36.02%) compared with that in the MTX group. These data reflect the dose-dependent effect of VC on the MDA level (Figure 2A). The pretreatment with 100 mg/kg of VC resulted in a significant increase in the SOD level (348.53%) compared with that in the MTX group. However, the pretreatment with 200 mg/kg of VC resulted in a more significant increase in the SOD level (542.54%) compared with that in the MTX group. The pretreatment with 100 mg/kg of VC resulted in a mild increase in the GSH levels (26.03%) compared with those in the MTX group, whereas the pretreatment with 200 mg/kg of VC resulted in marked increase (54.14%), indicating the dose-dependent effect of VC on combating oxidative stress.

The pretreatment of the mice with 10 mg/kg/day of CUR and 20 mg/kg/day of CUR orally for 10 days before the administration of a single dose of MTX resulted in the amelioration of hepatocyte oxidative stress induced by MTX through the reduction of the MDA level compared with that in the MTX group. This reduction was statistically significant with the percentage of the difference (53.307 and 57.86%) between both the doses (Figure 2A).

The SOD level was increased in the CUR-pretreated groups. This increase appeared to be approximately equal between the 2 doses of CUR. The SOD level was increased by 28.693% in the group pretreated with 10 mg/kg of CUR and by 31.39% in the group pretreated with 20 mg/kg of CUR compared with that in the MTX group, but these increases were statistically insignificant.

The pretreatment with 10 mg/kg of CUR resulted in a minor increase in the GSH level (13.25% in the MTX group), whereas the GSH level in group pretreated with 20 mg/kg of CUR was. This increase in both the CUR groups was statistically insignificant. When the percentage of the difference between 10 mg/kg/day of CUR (13.25%) and that of 20 mg/kg/day of CUR (16.757%) was compared, an obvious dose-dependent effect of CUR as observed on the GSH level (Figure 2C).

The pretreatment of the mice with 10 mg/kg/day of CUR and 100 mg/kg/day of VC for 10 days before the injection of single-dose MTX (20 mg/kg i.p.) on the tenth day resulted in the amelioration of hepatocyte oxidative stress induced by the single dose of MTX. This amelioration effect was manifested by the MDA level, which was significantly decreased (62.66%) compared with the MDA level in the MTX group, and the effect was more significant than that of VC and CUR alone.

The SOD level was significantly increased in the combination group (428.05%) compared with that in the MTX group, and the effect was more significant than that of VC and CUR alone. The GSH level was decreased in the pretreated combination group (53.35%) compared with that in the MTX group. This decrease was statistically more significant than that in the CUR group.

### Effects of Treatment on Liver Enzymes

The treatment of the mice with single-dose MTX (20 mg/kg i.p.) resulted in intervention with the liver enzymes, manifested by a significant increase in the serum level of ALT (250.11%) compared with that in the control group (Table 1).

**TABLE 1:** Changes in serum liver enzyme between mice treated with MTX, vitamin C, and curcumin alone and in combination (pretreatment groups).

Groups	Parameters assessed			
	ALT (IU/L)	AST (IU/L)	ALP (IU/L)	LDH (ng/mL)
Control	33.42 ± 1.92	27.42 ± 3.15	267.28 ± 15.63	21.83 ± 1.20
MTX	50.71 ± 3.67^a^	34.71 ± 2.78^a^	458 ± 34.91^a^	38.48 ± 3.62^a^
VC 100	30.28 ± 1.16^b^	30.85 ± 1.51	200.91 ± 21.01^b^	22.89 ± 1.53^b^
VC 200	26.00 ± 3.54^b^	26.14 ± 2.27^b^	126.00 ± 11.85^b^	18.40 ± 1.52^b^
CUR 10	43.38 ± 3.16	31.23 ± 3.51	315.91 ± 31.21^b^	12.7 ± 2.496^b^
CUR 20	36.00 ± 2.54^b^	28.34 ± 2.16^b^	256.00 ± 13.5^b^	11.66 ± 3.22^b^
VC100 + CUR 10	27.63 ± 3.64^bd^	27.48 ± 3.5^b^	188.32 ± 18.5^bcd^	10.82 ± 1.22^bc^

*Data are expressed as mean ± standard deviation (n = 7 per group). Significant difference vs. ^a^respective control saline, ^b^respective MTX group, ^c^respective 100 mg/kg vitamin C group, ^d^respective 10 mg/kg curcumin group, each at p<0.05.*

MTX induced a significant increase in the levels of AST, ALP, and LDH (26.58, 71.33, and 76.27%, respectively) compared with the control group.

The pretreatment of the mice with 100 and 200 mg/kg/day of VC (*n* = 7) was orally administered for 10 days before the administration of a single dose of MTX on the tenth day, ameliorating the damaging effect of MTX on hepatocytes compared with that in the MTX group. This amelioration effect was reflected by a significant decrease in the ALT serum levels (40.28 and 48.71%) in mice that received 100 and 200 mg/kg/day of VC, respectively. This comparison reflects the dose-dependent effect of VC on the ALT serum level (Table 1). The AST serum level was insignificantly decreased (11.12%) in the group pretreated with 100 mg/kg/day of VC, whereas the increased dose of up to 200 mg kg/day induced a more significant decrease of 24.69% compared with that in the MTX group.

A significant and dose-dependent effect of VC on the ALP serum level was demonstrated by the percentage of the difference between the groups pretreated with 100 (56.13%) and 200 (72.489%) mg/kg of VC. The LDH serum level of the groups pretreated with 100 and 200 mg/kg/day of VC was significantly decreased (40.51 and 52.18%, respectively) compared with that of the MTX group. The pretreatment with 10 mg/kg/day of CUR caused a mild decrease in the ALT serum level (14.45%) compared with that in the MTX-treated group; this decrease was statistically insignificant. However, the pretreatment with 20 mg/kg/day of CUR resulted in a more significant reduction in the ALT serum level (29%) compared with that in the MTX group. The pretreatment with 10 mg/kg/day of CUR resulted in a mild decrease in the AST level (10.02%), and this decrease was statistically insignificant. However, the pretreatment with 20 mg/kg/day of CUR reflected a significant decrease in the AST serum level compared with that in the MTX group (18.35%). Both the doses of CUR (10 and 20 mg/kg/day) led to a significant decrease in the ALP serum level (31 and 44%, respectively) compared with that in the MTX group.

The LDH serum level with 10 mg/kg/day of CUR pretreatment was significantly decreased 66.69%) compared with that in the MTX group. However, the LDH serum level with 20 mg/kg/day of CUR was significantly decreased with the percentage of difference (69.659%). These data may reflect a dose-dependent effect of CUR on the LDH serum level during MTX-induced hepatotoxicity (Table 1).

The pretreatment of the mice with 10 mg/kg/day of CUR and 100 mg/kg/day of VC for 10 days before the injection of a single dose of MTX on the tenth day resulted in pronounced hepatoprotective effects. This effect was manifested by the decreased ALT serum level (45.51%) compared with those in the MTX group. However, the AST serum level was slightly decreased compared with that in the MTX group (20.82%). This decrease was statistically significant.

The combination therapy induced a significant decrease in the ALP serum levels (58.88%) compared with the levels in the MTX group, and the effect was more significant than a single treatment. The LDH serum level was also significantly decreased (71.88%) compared with that in the MTX group (Table 1).

### Liver Histopathological Findings

**FIGURE 3:**
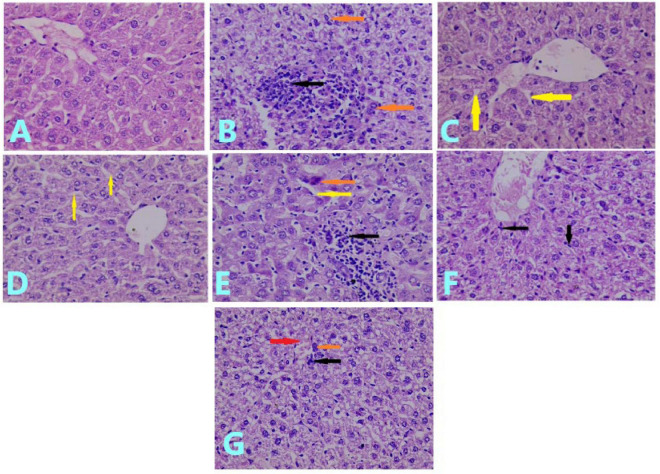
Effects of treatments on histopathological examination of the liver tissue: **(A)** liver section of control group showed normal hepatocytes. (H&E; ×40). **(B)** Liver section of MTX-treated group showed necrosis of hepatocytes (brown arrow), infiltration of the inflammatory cell (black arrow), and prominent cellular degeneration (H&E; ×40). **(C)** Liver section of mice pretreated with 100 mg/kg of VC showed slight sinusoidal dilation (arrow) with mild cellular degeneration (H&E; ×40). **(D)** Liver section of the group pretreated with 200 mg/kg of VC showed slight sinusoidal dilation (yellow arrow) (H&E; ×40). **(E)** Liver section of the group pretreated with 10 mg/kg of CUR showed hepatocyte necrosis (brown arrow), sinusoidal dilation (yellow arrow), and inflammatory cell infiltration (black arrow) (H&E; ×40). **(F)** Liver section of the group pretreated with 20 mg/kg of CUR showed cellular degeneration with mild infiltration of inflammatory cells (black arrow) (H&E; (×40). **(G)** Liver section of mice pretreated with VC and CUR showed slight inflammatory cell infiltration (black arrow), necrotic cells (brown arrow), and slight congestion (red arrow) (H&E; ×40).

The histopathological examination of the liver tissue of the control group showed normal hepatocytes (Figure 3A), whereas the MTX group showed severe liver injury, represented by the necrosis of hepatocytes, infiltration of inflammatory cells, and prominent cellular degeneration. The injury represented about 65% of the field (compared to Figure 3A), which was scored as +++ (Figure 3B).

The pretreatment with 100 mg/kg of VC for 10 days before the administration of MTX showed a protective effect on the liver tissue, for which the histopathological examination showed slight sinusoidal dilation with mild degeneration of hepatocytes. This was observed in < 5% of the field and was scored as ± (Figure 3C). The histopathological examination of the liver of the mice pretreated with 200 mg/kg of VC showed slight dilation of a sinusoid inside hepatocytes. This occupied < 5% of the field and was scored as ± (Figure 3D). The histopathological examination of the liver of the group pretreated with 10 mg/kg of CUR for 10 days before the administration of MTX showed mild cellular degeneration of hepatocytes, necrosis of hepatocytes, infiltration of inflammatory cells, and sinusoidal dilation. The injury occupied 45% of the field, which was scored as + + (Figure 3E).

The histopathological examination of the group pretreated with 20 mg/kg of CUR showed cellular degeneration and less infiltration of inflammatory cells than the previous group, and the injury occupied 30% of the field, with a score of + + (Figure 3F).

The pretreatment with 10 mg/kg of CUR and 100 mg/kg of VC for 10 days before the administration of MTX resulted in a better protective effect on the liver tissue than the effect of curcumin alone. The histopathological examination of the liver of this group showed slight congestion, cellular degeneration of hepatocytes, scant necrotic cells, and slight inflammatory cell infiltration. The injury occupied 18% of the field, with a score of + (Figure 3G).

## Discussion

The results of the present study revealed that MTX induces liver damage via increased oxidative stress in the liver tissue and that pretreatment with CUR and/or VC produces a hepatoprotective effect through the modulation of oxidative stress biomarkers.

In the current study, MTX produced an oxidative stress status, as demonstrated by the decreased GSH and SOD levels and increased MDA level, which is in agreement with previous studies (9, 24, 25). The significant depletion of GSH caused by MTX decreased the antioxidant defense mechanism of the cell, thus sensitizing the cell to oxidative stress (26).

The generation of ROS along with the depletion of the cellular antioxidant defense mechanism resulted in increased lipid peroxidation, as demonstrated by the significant increase in the MDA tissue level in the MTX-treated mice compared with that in the control group in the present study.

The treatment of mice with a single dose of MTX resulted in a significant increase in the ALT, LDH, and ALP levels and an increased in the AST level compared with those in the control group. This increase agrees with those of other studies (6, 7, 24).

In the present study, the pretreatment with different doses of VC (100 and 200 mg/kg/day) resulted in the attenuation of liver injury produced by MTX. This attenuation was demonstrated by the significantly decreased serum levels of ALT, ALP, and LDH, along with the decreased AST level, which reflects the hepatoprotective effect of VC. The pretreatment with VC also resulted in increased tissue levels of SOD and GSH and a decreased level of MDA, which agrees with a previous finding (27).

The decreased levels of ALT, AST, and MDA in the present study agree with previous findings that represented the protective effect of VC on the liver of carbon tetrachloride-intoxicated rats (28, 29). Several studies have reported that VC produces a protective effect against drugs and chemical agents that induce hepatotoxicity (28, 29). The target of VC is the mitochondria, preventing mitochondrial swelling, mitochondrial membrane potential dissipation, and ROS burst, thereby preventing hepatic apoptosis; these effects may oppose the action of MTX (15, 20). As a result, VC can neutralize ROS or reactive nitrogen species produced by MTX in the hepatocyte, decrease their availability to interact with intracellular organelles, and prevent the initiation of lipid peroxidation. Thus, pretreatment with VC prevents hepatocyte damage and the leakage of cytosolic enzymes, as demonstrated in the present study by the decreased serum ALT, AST, ALP, and LDH levels when the VC group was compared with the MTX group.

The MDA tissue level decreased with VC pretreatment in the present study. The biochemical results showed the dose-dependent effect of VC on the production of its hepatoprotective effect against MTX-induced hepatotoxicity, represented by the dose-dependent decrease in the MDA tissue level. This result implied less lipid peroxidation and a dose-dependent decrease in the serum ALT and ALP levels, which reflected the reduced damage of hepatocytes and bile duct membrane or bile duct epithelial cell. The dose-dependent effect of VC reflected by the present study is in agreement with that of a previous study, in which the antioxidant mechanism of VC was documented as dose dependent (17).

The protective effect of CUR against MTX-induced hepatotoxicity was evaluated in the present study. The pretreatment of the mice with different doses of CUR resulted in the attenuation of hepatotoxicity induced by MTX, represented by the significant decrease in the tissue MDA level and increase in the SOD and GSH tissue levels. These results agree with those of a previous study, which investigated the hepatoprotective effect of phytosome CUR on paracetamol-induced hepatotoxicity (30). The decreased tissue level of MDA in the present study also agrees with another study that investigated the hepatoprotective effect of CUR on MTX-induced hepatotoxicity (11).

It has been reported that CUR increases the concentration of GSH and the activity of GSH peroxidase and SOD enzymes through upregulation of *Nrf2* genes (31, 32). CUR also increases the GSH level through a pro-oxidant mechanism that can induce a GSH-antioxidant response and, thus, provide hepatic protection (33). It has also been reported that CUR causes indirect reduction of ROS via a decrease in the O_2_ level and downregulation of the expression of some nicotinamide adenine dinucleotide phosphate oxidase subunits (33).

In addition, CUR has anti-inflammatory, antioxidant, antimicrobial, anticancer effects through inhibition release of pro-inflammatory cytokines, scavenging of ROS and inhibition proliferation of different microbes, respectively (34). CUR components mainly turmerone, furanodiene, bisacurone, curdione, germacone, calebin A and cyclocurcumin have strong anti-inflammatory effects (35). Therefore, in virtue of its anti-inflammatory and antioxidant effects CUR can reduce MXT-induce acute liver injury.

*In vitro* study illustrated that CUR in combination with quercetin produced significant antibacterial and anti-inflammatory activities by inhibiting expression of cyclooxygenase (COX-2), NF-κB and nitric oxide (NO) (36). Experimental study by Alhusaini et al. demonstrated that CUR in combination with VC was effective in the attenuation of Lead-induced hepatotoxicity through inhibition of DNA damage, oxidative stress and NF-κB signaling pathway (37). Combination of MTX with CUR was used as anticancer therapy by an additive cytotoxicity effect through modulation of different phases of cell-cycle (38). Moghadam et al. found that pre-treatment with CUR can prevent MTX-induced acute hepatic injury by inhibiting expression of inflammatory and ROS genes, thereby decreasing oxidative and inflammatory-mediated acute hepatic injury (39). Besides, VC has cytoprotective effects against structural and functional changes induced by MTX by reducing expression of ROS and progression of oxidative stress in MTX-induced hepatotoxicity (40).

The pretreatment with different doses of CUR in the present study resulted in a significant dose-dependent decrease in the LDH, ALT, AST, and ALP serum levels. The results of ALT, AST, and ALP also agree with those of a previous study (11). CUR might mediate its hepatoprotective effect through multiple mechanisms, thus possibly modulating the hepatocyte apoptosis process. The significant decrease in the serum LDH level in the CUR-pretreated group, along with the dose-dependent decrease in the ALT level, which is hepatocyte-specific, reflected the hepatoprotective effect of CUR against MTX-induced hepatotoxicity through decreasing oxidative stress in hepatocytes, thus preventing the leakage of the intracellular enzymes into the serum (9).

CUR exerts an anti-inflammatory action by decreasing the expression and secretion of proinflammatory cytokines and suppresses the activation of NF-κB (16). MTX produces hepatotoxicity through stimulation of NF-κB in Kupffer cells via ROS, with subsequent release of proinflammatory cytokines, mainly TNF-α (7, 41). Thus, CUR can produce a hepatoprotective effect through its anti-inflammatory mechanism.

The pretreatment with 100 mg/kg/day of VC and 10 mg/kg/day of CUR resulted in a significant decrease in the MDA tissue level and an increase in the SOD and GSH levels compared with those in the MTX-treated group.

The significant decrease in the MDA level in the present study was in agreement with a previous study that investigated the hepatoprotective effect of CUR and VC on cadmium-induced hepatotoxicity (42). The combined effect reflected reduction in oxidative stress in the presence of MTX-induced hepatotoxicity, as demonstrated by a significant decrease in the MDA tissue level and augmentation of cellular antioxidant mechanisms, reflected by increased SOD% and GSH levels.

The beneficial effect of the combination was reflected mainly by MDA, SOD, and GSH, and this finding may be related to multiple mechanisms of action of CUR in reducing lipid peroxidation and antioxidant mechanisms.

The present study showed the amelioration of liver injury induced by MTX via pretreatment with 100 and 10 mg/kg/day of VC and CUR, respectively. This result was demonstrated by the decrease in the ALT, AST, and ALP levels, along with a significant decrease in the LDH serum levels.

The amelioration of oxidative stress induced by MTX by the combined pretreatment explains the decrease in liver enzymes. Attenuated oxidative stress resulted in less damage to hepatocyte organelles and membrane, indicating less leakage of the cytosolic enzyme into the extracellular part and, subsequently, less detected in the serum (9).

The histopathological examination of the liver of MTX-treated mice supported the biochemical results by presenting a marked hepatic injury compared with that in the control group. This finding showed that severe injury is represented by necrosis of hepatocytes, infiltration of inflammatory cells, and prominent depletion of glycoprotein, in agreement with a previous study (43). The pretreatment with VC reflected the hepatoprotective effect, as determined in the histopathological examination, which supported the biochemical results. The pretreatment with 100 and 200 mg/kg/day resulted in very mild hepatic injury, represented by slight sinusoidal dilation along with mild depletion of hepatocytic glycoprotein.

The histopathological examination of the liver of the mice pretreated with 10 mg/kg/day of CUR before the administration of MTX reflected a hepatoprotective effect. However, the histopathological examination of the mice pretreated with 20 mg/kg/day of CUR showed more depletion of glycoprotein and less infiltration of inflammatory cells than the 10 mg/kg-pretreated group.

The histopathological examination reflected an obvious hepatoprotective effect of the combined treatment compared with the MTX group, which indicated the synergistic effect of VC and CUR when used together as a pretreatment. There was slight congestion, depletion of glycoprotein granules inside the hepatocytes, fatty material accumulation with scant necrotic cells, and a few inflammatory cell infiltrations. The injury occupied only 18% of the field.

The present experimental study had several limitations including small sample size, analysis of total liver ROS gene expression was not performed, sex hormone portion which may be affected by MTX-induced hepatotoxicity and by used drugs in this study. Likewise, different magnification of histo-pathological slides was not performed; we only used H&E × 40 which may affect the microscopic findings. In addition, a gender difference was not evaluated as we used female mice only. Despite of these limitations, the present study is regarded as a preliminary step for large-scale clinical study to evaluate the hepatoprotective of CUR, VC and their combination against MTX-induced hepatotoxicity mainly in patients with chronic use of MTX as in rheumatoid arthritis and psoriasis.

## Conclusion

MTX-induced hepatotoxicity is mediated by induction of oxidative stress as evident by increase biomarkers of lipid peroxidation and reduction of antioxidant enzyme activity. Pretreatment with VC, CUR or their combination reduce development of MTX-induced hepatotoxicity by antioxidant and anti-inflammatory effects. The combined effect of VC and CUR provided a synergistic hepatoprotective effect than the pretreatment with low dose of VC 100 mg/kg or CUR alone. However, this combined effect produced nearly similar effect to that of large dose of VC 200 mg/kg. In this regards, preclinical and clinical studies are warranted to confirm the combined hepatoprotective effect of VC and CUR against MTX-induced hepatotoxicity.

## Data Availability Statement

The raw data supporting the conclusions of this article will be made available by the authors, without undue reservation.

## Ethics Statement

The animal study was reviewed and approved by the Serum and Vaccine Institute and the National Center for Drug Control and Research.

## Author Contributions

DH, AA-G, HA-K, AE-K, EE, WN, SS, SC, AT, SA, and GB performed methodology, analysis, data interpretation, data curation, statistical analysis, revision, and literature review. All authors listed have made a substantial, direct, and intellectual contribution to the work, and approved it for publication.

## Conflict of Interest

The authors declare that the research was conducted in the absence of any commercial or financial relationships that could be construed as a potential conflict of interest.

## Publisher’s Note

All claims expressed in this article are solely those of the authors and do not necessarily represent those of their affiliated organizations, or those of the publisher, the editors and the reviewers. Any product that may be evaluated in this article, or claim that may be made by its manufacturer, is not guaranteed or endorsed by the publisher.
